# The Effects of Physical and Mental Fatigue on Time Perception

**DOI:** 10.3390/sports12020059

**Published:** 2024-02-15

**Authors:** Reza Goudini, Ali Zahiri, Shahab Alizadeh, Benjamin Drury, Saman Hadjizadeh Anvar, Abdolhamid Daneshjoo, David G. Behm

**Affiliations:** 1 School of Human Kinetics and Recreation, Memorial University of Newfoundland, St. John’s, NL A1C 5S7, Canada; rgoudini@mun.ca (R.G.); azahiri@mun.ca (A.Z.); shadjizadeha@mun.ca (S.H.A.); 2 Faculty of Kinesiology, University of Calgary, Calgary, AB T2N 1N4, Canada; shahab.a91@gmail.com; 3 Sport & Exercise Science, Hartpury University, Gloucestershire GL19 3BE, UK; ben.drury@hartpury.ac.uk; 4 Department of Sport Injuries and Corrective Exercises, Faculty of Sport Sciences, Shahid Bahonar University of Kerman, Kerman 76169-13439, Iran; daneshjoo.hamid@gmail.com

**Keywords:** temporal, time estimate, Stroop task, heart rate, rating of perceived exertion

## Abstract

The perception of time holds a foundational significance regarding how we elucidate the chronological progression of events. While some studies have examined exercise effects on time perception during exercise periods, there are no studies investigating the effects of exercise fatigue on time perception after an exercise intervention. This study investigated the effects of physical and mental fatigue on time estimates over 30 s immediately post-exercise and 6 min post-test. Seventeen volunteers were subjected to three conditions: physical fatigue, mental fatigue, and control. All participants completed a familiarization session and were subjected to three 30 min experimental conditions (control, physical fatigue (cycling at 65% peak power output), and mental fatigue (Stroop task)) on separate days. Time perception, heart rate, and body temperature were recorded pre-test; at the start of the test; 5, 10, 20, 30 seconds into the interventions; post-test; and at the 6 min follow-up. Rating of perceived exertion (RPE) was recorded four times during the intervention. Physical fatigue resulted in a significant (*p* = 0.001) underestimation of time compared to mental fatigue and control conditions at the post-test and follow-up, with no significant differences between mental fatigue and control conditions. Heart rate, body temperature, and RPE were significantly (all *p* = 0.001) higher with physical fatigue compared to mental fatigue and control conditions during the intervention and post-test. This study demonstrated that cycling-induced fatigue led to time underestimation compared to mental fatigue and control conditions. It is crucial to consider that physical fatigue has the potential to lengthen an individual’s perception of time estimates in sports or work environments.

## 1. Introduction

People have been fascinated by time for centuries; however, philosophers and scientists from ancient to modern times have yet to fully agree on its definition and qualities [1]. The concept of time is one of the experiences that are essential for how we experience the world [2]. Our behavioral and cognitive systems depend heavily on duration perception, which allows us to interact with the outside world [3]. An accurate perception of time is an indispensable part of many time-constrained sports (i.e., North American football, basketball, figure skating, and others) and work environments [4]. It is well known that our subjective perception of time can be manipulated and distorted under certain circumstances [5]; however, little is known about how physical and mental fatigue affects how people perceive time.

There are two prominent theories pertaining to time perception: the Pacemaker–Accumulator Model (PAM), alternatively referred to as the Scalar Expectancy Theory (SET) [6], and the Striatal Beat Frequency Model (SB-FM) [7,8,9]. Both theoretical frameworks elucidate that time perception is significantly impacted by arousal [7,10]. The Scalar Expectancy Theory uses a clock, memory, and decision stages to divide the temporal processing system. The SB-FM not only discusses the timing behaviors but also identifies which neural regions of the brain are involved [11]. The model suggests that time estimation is determined by dopamine and glutamate activity near the substantia nigra and ventral tegmental area that monitor activation patterns in the cortex’s oscillatory neurons, which are controlled by glutamate action [12]. The oscillating rate of neurons synchronizes when an interval starts, and the spiny neurons are reset by phasic dopaminergic input. A dopamine pulse is released when the target duration is attained, strengthening the synapses [12]. Once the same signal duration has been timed once more, neostriatal GABAergic spiny neurons compare the current activation pattern to the stored pattern to determine when the duration has been reached [11,12,13]. Exercise modulates dopamine and glutamate neurotransmission, increasing cerebral blood flow, with different exercise types differentially affecting frontal–striatal-related circuits. Hence under this model, exercise could affect time perception [14].

Arousal associated with physical and mental activity can increase heart rate, muscle activation (e.g., motor unit recruitment and firing frequency), thermoregulation, and other physiological or external signals, which have the potential to alter time perception [15]. The increased activity gives rise to additional events within the temporal processing system, leading to an accelerated perception of time in response to higher-intensity contractions. Given the cerebellum’s involvement in both movement and temporal processing [16], exercise-induced arousal may exert more influence on time perception compared to other forms of arousal. The heightened demands in terms of frequency of events during sensory afferent processing may also play a role in impacting time perception [15]. Exercise encompasses a wide range of forms, with differences in intensity, duration, and movement types. Previous studies have shown that continuous low–moderate-intensity exercise frequently leads to greater central fatigue [17,18,19,20] and, thus, is more likely to affect time perception than shorter-duration high-intensity fatigue. However, time perception research often overlooks the significance of contraction types, such as dynamic contractions. Additionally, the impact of varying fatigue protocols on time perception remains relatively unexplored, representing an understudied factor that could potentially affect time perception positively or negatively.

As a psychobiological condition, mental fatigue (e.g., difficulty in maintaining focus, attention, and cortical excitability) results from extended periods of demanding cognitive activity [21]. It has been shown that mental fatigue increases prefrontal cortex activation, cerebral perfusion, and somatomotor activation. This increase in blood flow to the prefrontal cortex may result from additional neural activation required to produce efferent motor commands, which may be one of the reasons why central fatigue can have an impact on physical performance [22,23]. After finishing a mentally exhausting task, an approach is to look at behavioral performance deficiencies on a subsequent task [24]. The best explanation for behavioral changes during such tasks is a reduction in top-down processing, which results in an inability to focus and meet task demands.

Exercising for a long time in a hot environment impairs physical and mental performance [25]. Timing behavior is sensitive to changes in body temperature; hence, it has been suggested that there is a temperature-sensitive time mechanism [26]. Some studies claim that a rise in temperature causes time to hasten, but others contend that this effect only happens once a certain threshold of perceived fatigue has been reached [26,27].

Since no studies have compared physical and mental fatigue in relation to time perception, the objective of this study was to compare the effects of physical (exhaustive cycling exercise protocol) and mental fatigue (Stroop task test) on time perception. It was hypothesized that physical and mental fatigue protocols would lead to an underestimation of the time.

## 2. Methods

### 2.1. Participants

An “a priori” statistical power analysis (software package, G * Power 3.1.9.7) was conducted based on the time perception of related studies [28] to achieve an alpha of 0.05, an effect size of 0.4, and a statistical power of 0.8 using the F-test family. The analysis indicated that between 12–14 participants per condition was sufficient to achieve adequate statistical power. Seventeen (17) healthy and recreationally active participants voluntarily took part in this study (Table 1). Activity levels were based on a self-report with recreationally active defined as individuals who participated in any kind of physical activity for a minimum of 2 days per week over the preceding 6 months. The exclusion criteria included the following: participants who have neurological conditions, knee injuries, the presence of medical issues that prevent high-intensity exercise, or injuries to the quadriceps muscles that could affect pedaling. The inclusion criteria included that participants need to be healthy and recreationally active. The recruitment period for participants began on 1 April 2023 and was completed by 8 September 2023.

Prior to their lab visit, participants were given instructions to avoid intense activity (24 h prior to participating) and to stop drinking alcohol, smoking, and using caffeine (12 h prior to participating). Each participant completed the physical activity readiness questionnaire plus (PAR-Q+ 2020), read and signed the informed consent form prior to testing and after a brief explanation of the study and the experiment’s procedures. Informed consent was obtained from all subjects involved in the study. During their first visit to the lab, every participant became familiar with all psychological measurements. The Institutional Health Research Ethics Board (ICEHR #20231533-HK) gave its approval for this study, which was carried out in accordance with the most recent version of the Helsinki Declaration.

### 2.2. Experimental Design

**Figure 1:**
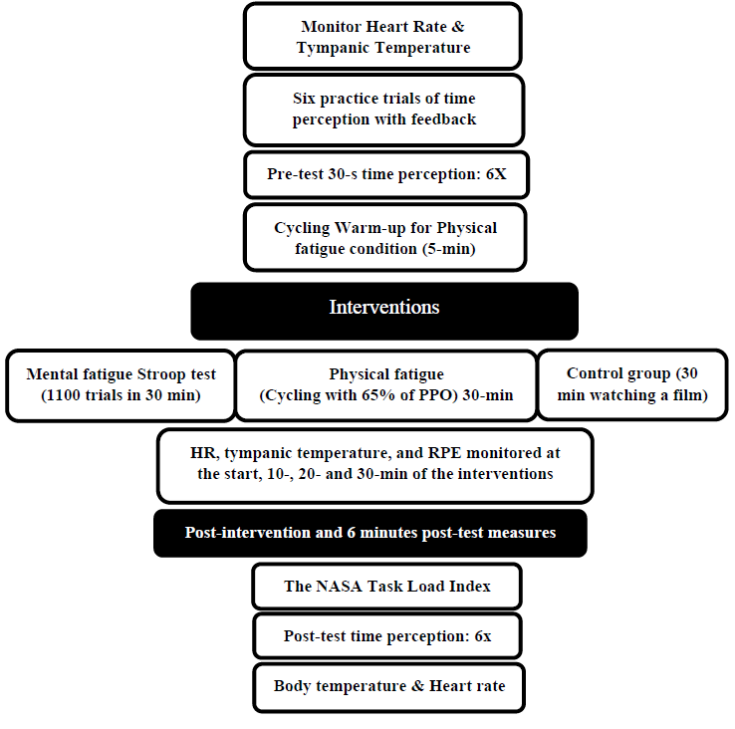
Experimental design. PPO: peak power output, RPE: Rating of Perceived Exertion.

The effects of 30 min of physical and mental fatigue on time perception were investigated using a randomized crossover study design. The participants became familiar with a basic orientation to the testing procedures and equipment during the initial familiarization session, and they performed an incremental cycling test using a Velotron ergometer (Velotron RacerMate, Seattle, WA, USA) to determine their peak power output (PPO). The participants then came to the lab for three distinct testing sessions: physical fatigue (30 min of cycling at 65% of peak power output), mental fatigue (1100 trials of a Stroop test with a duration of 30 min), or control (30 min watching a film). The time perception testing involving estimating the time lapse of 5, 10, 20, and 30 s (6 trials for each test) occurred at pre-test, immediate post-intervention, and 6 min post-intervention. In addition, heart rate, tympanic temperature, and ratings of perceived exertion were obtained at 10, 20, and 30 min into the intervention. Each session was randomized and separated by at least 48 h (Figure 1).

## 3. Dependent Variables: Measures

### 3.1. Time Perception

Prior to the intervention, participants watched a digital clock count to 30 s, twice, followed by four trials of time estimate practice of 30 s duration (estimate 5, 10, 20, and 30 s) with feedback. Then, for the pre-test data collection participants sat in a chair to estimate the time intervals of 5, 10, 20, and 30 s, six times without feedback. We chose to execute the intervals six times as individuals can ingrain this into memory (as stated by the Scalar Expectancy Theory). Thirty seconds was chosen as this approximate time restriction is common in a number of sports including basketball, tennis, North American football, and others. This procedure has also been used successfully in prior experiments conducted in this lab with intraclass correlation coefficients (ICCs) of 0.75–0.85 [15,29]. In the present study, a high degree of reliability was found between time perception measurements, with an ICC of 0.802 and a 95% confidence interval from 0.628 to 0.916 (F^(16,176)^ = 5.058, *p* < 0.001). With the six 30 s time estimate attempts for each testing time, the mean scores were analyzed for the pre-test, immediately post-test, and 6 min follow-up. Since six 30 s time estimates equals 3 min, we decided to be consistent and permit a 3 min recovery before the next 3 min testing period (6 min in total). To estimate time, a hand dynamometer (custom-built design) connected to a BioPac AcqKnowledge data acquisition system (Goleta, CA, USA) was used.

### 3.2. Heart Rate and Tympanic Temperature

Heart rate (T31, Polar, Kempele, Finland) and tympanic temperature (IRT6520CA ThermoScan, Braun, Germany) were monitored and recorded in the pre-test, during the experimental protocols (start, 10, 20, and 30 min), and at the post-test. A heart rate monitor was fixed using an elastic belt secured around the participant’s sternum. Tympanic temperature was acquired with a thermometer probe, fitted with a disposable plastic covering, which was gently inserted into the right ear canal.

### 3.3. Rate of Perceived Exertion (RPE)

The RPE Borg Scale [30] was used as a tool for assessing the intensity of participants’ activity during the intervention, utilizing a graduated scale ranging from 6 to 20. Throughout the physical fatigue, mental fatigue, and control condition experiments, participants were verbally prompted to provide their RPE ratings. The main aim of using the RPE Borg Scale was to gain valuable insights into whether participants were engaging in the prescribed activity at the desired intensity [15].

### 3.4. NASA-TLX

The NASA-TLX was implemented for all three conditions. It is a tool for measuring mental workload that aims to record workers’ subjective perceptions of complex socio-technical systems that involve humans and machines. Due to its multidimensional nature and ease of administration, the NASA-TLX is perhaps the most commonly used mental workload scale [31]. NASA-TLX has six subscales that measure mental demand, physical demand, temporal demand, performance, effort, and the level of frustration [32]. Following the implementation of interventions in each experimental condition, participants manually completed the NASA-TLX questionnaire. Participants were required to rate each item using a scale consisting of 20 equidistant intervals delineated by bipolar descriptors (e.g., high/low). Subsequently, the computed score was scaled by a factor of 5, yielding a resultant score ranging from 0 to 100 for each of the subscales [33].

### 3.5. Independent Variable: Protocol

Prior to the pre-test, during the intervention (start, 10, 20, and 30 min), after the time perception test, and 6 min after the time perception test (follow-up), participants’ tympanic temperatures and heart rates were recorded. The RPE was also recorded at the start and 10, 20, and 30 min into the intervention for the three conditions.

### 3.6. Maximal Incremental Cycling Test Protocol

The maximum cycling exercise protocol was used to determine the maximum wattage (W_max_) for the incremental test on a cycle ergometer (Velotron RacerMate, Seattle, WA, USA). Each participant’s ideal seat height on the cycle ergometer was determined, recorded, and used for the following sessions. Participants warmed up at 59 watts with an RPM of 70 for 5 min; then, participants began cycling at 80 watts for 3 min with an RPM of 70, and the resistance was increased by 40 watts every 3 min until they reached exhaustion (a cadence of less than 60 RPM for more than 5 s, despite intense verbal encouragement). The researcher verbally encouraged participants during the test to perform a true all-out effort. The W_max_ (i.e., peak power output (PPO)) was calculated with the formula: W_max_ = W_out_ + (t/180) × 40 [W_out_: workload of the last completed stage; t: time (seconds) in the final stage] [33].

### 3.7. Exhaustive Cycling Exercise Protocol

Following the orientation practice time estimate sessions (two observations of a clock showing 30 s followed by four estimates of 30 s with feedback) and pre-tests, participants warmed up on the cycle ergometer for 5 min (at 59 watts) and then cycled at 65% PPO for 30 min. On the cycle ergometer, participants’ positions were adjusted to replicate their maximum cycling exercise. After the exhaustive cycling exercise protocol, participants filled out the NASA Task Load Index and were then tested with six time estimate trials immediately as well as six minutes after the immediate post-test time estimates.

### 3.8. Stroop Task

The Stroop color–word test, a widely used neuropsychological test, measures a subject’s capacity to suppress cognitive interference, which happens when the processing of one stimulus attribute interferes with the concurrent processing of another [34]. It has been demonstrated that the Stroop task, which demands prolonged attention and response inhibition, induces a state of mental fatigue [35]. The participants were asked to identify the color of a word without regard for its actual meaning. Fifty percent (50%) of the trials were congruent (matched word and color), whereas 50% were incongruent, according to a pseudo-random sequence that was used to govern the trials (with all incongruent word–color combinations). The participants were then instructed to push a key on a keyboard that matched the color of a text displayed on a screen. The computer screen was 33 cm, and all participants used this laptop to observe the 30 s time estimate and Stroop tasks throughout the study. For 1000 ms, each word appeared on the screen in a font size of 34, and then, the screen remained blank before the next word appeared [33]. In this investigation, we conducted a total of 1100 trials to induce mental fatigue, requiring an approximate duration of 30 min for its completion. Slimani et al. (2018), in their study, showed that successfully performing the Stroop task for 30 min induced mental fatigue [36]. For online data collection (Stroop task test), the software PsyToolkit (Version 3.4.4) was used [37,38].

### 3.9. Control Condition

The control condition executed the six trials (pre-test), watched a documentary film “*When We Left Earth: The NASA Missions—Episode 6: A Home in Space*” (Discovery Channel, New York, NY, USA) for 30 min [33], and then filled out the NASA Task Load Index (NASA-TLX).

## 4. Statistical Analysis

Statistical analyses were calculated using SPSS software (version 28.0, SPSS, Inc., Chicago, IL, USA). The Shapiro–Wilk and Mauchly’s tests were used to assess the normality of the distribution and assumption of sphericity, respectively (*p* > 0.05). The data for time perception were analyzed using the means of six trials. A repeated measures analysis of variance (ANOVA) including three testing times (pre-test, post-test, and 6 min follow-up) and three conditions (control, mental, and physical fatigue) was conducted to determine significant differences in time perception for each time estimate (5, 10, 20, and 30 s) separately (within time estimate analysis). One-way repeated measures were conducted to determine significant differences between testing times (pre-test, post-test, and follow-up). A repeated measures ANOVA including three conditions (control, mental, and physical fatigue) and four time estimates (5, 10, 20, and 30 s) was conducted to determine significant differences between time estimates. The mean difference (MD) (which measures the absolute difference between the mean value in two groups) was used for the analysis, and it estimates the amount by which the experimental intervention changes the outcome on average compared with another condition. To analyze body temperature and heart rate, an ANOVA with repeated measures was used for seven testing times (pre-test; start; 10, 20, and 30 min; post-test; and follow-up) and three conditions (control, physical, and mental fatigue). To examine RPE during the intervention, a repeated measures ANOVA including four testing times (start, 10, 20, and 30 min) and three conditions (control, mental, and physical fatigue) was used. To analyze the NASA Task Load Index, a one-way repeated measures ANOVA was used for mental and physical demand subscales. If the interactions were significant, a Bonferroni post hoc test was conducted to detect the significant differences between conditions for each test. The effect sizes of each variable were tested using partial eta squared (η_p_^2^) (0.01 = small effect, 0.06 = medium effect, 0.14 = large effect). The statistical significance level was set at *p* < 0.05. Cohen’s d effect sizes were calculated for individual post hoc comparisons, with effect sizes such as trivial (d = <0.2), small (0.2–≤0.5), medium (d = 0.5–≤0.8), and large (d = ≥0.8) [39].

## 5. Results

### 5.1. Time Estimates

#### 5.1.1. Five Seconds

The results of the 5 s time estimates revealed a significant main effect for the conditions (F_(1.63,26.11)_ =8.44, *p* = 0.003, η_p_^2^ = 0.346), as well as an interaction between testing times and conditions (F_(4,64)_ = 10.08, *p* = 0.001, η_p_^2^ = 0.387). However, there was no significant main effect for the testing times (F_(2,32)_ = 3.20, *p* = 0.054, η_p_^2^ = 0.167). There were no significant differences in the interaction between condition and testing time among conditions at the pre-test, but there was a significant, large-magnitude underestimation of time for the physical fatigue condition compared to the mental fatigue (MD = −0.706 s, *p* < 0.001, d = 1.25) and control (MD = −0.577, *p* < 0.001, d = 1.45) conditions at the post-test and follow-up (mental fatigue (MD = −0.842 s, *p* < 0.001, d = 1.59) and control (MD = −0.698 s, *p* < 0.001, d = 1.71)). While physical fatigue resulted in an underestimation of time at the post-test and follow-up compared to mental and control conditions, the underestimation due to physical fatigue at the post-test (MD = −0.539 s) and follow-up (MD = −0.590 s) was also significantly greater than the pre-test, but there was no significant difference between the post-test and follow-up. Additionally, there were no significant differences between the mental fatigue and control conditions at the pre-test, post-test, and follow-up. The significant main effect for the conditions showed that there was an underestimation of time due to physical fatigue compared to mental fatigue (*p* < 0.011, MD = −0.500 s) and control (*p* < 0.001, MD = −0.469 s) conditions. There were no significant differences between mental fatigue and control conditions (Table 2).

#### 5.1.2. Ten Seconds

There were significant main effects with the 10 s time estimates for the condition (F_(1.40,22.39)_ =12.57, *p* = 0.001, η_p_^2^ = 0.440) and testing time (F_(2,32)_ = 4.75, *p* = 0.016, η_p_^2^ = 0.229), as well as a significant interaction between testing time and conditions (F_(4,64)_ = 16.91, *p* = 0.001, η_p_^2^ = 0.514). The interaction between condition and testing time showed that there were no significant differences at the pre-test, but there was a significant, large-magnitude underestimation of time with the physical fatigue condition compared to the mental fatigue (MD = −1.612 s, *p* < 0.001, d = 1.78) and control (MD = −1.366 s, *p* < 0.001, d = 2.0) conditions at the post-test, as well as with the mental fatigue (MD = −2.067 s, *p* < 0.001, d = 1.90) and control (MD = −1.609 s, *p* < 0.001, d = 1.95) conditions at the follow-up. There were no significant differences between mental fatigue and control conditions at the pre-test, post-test, and follow-up. There was a significant (main effect for conditions) underestimation of time due to the physical fatigue condition compared to mental fatigue (*p* = 0.001, MD = −1.246 s) and control (*p* < 0.001, MD = −1.077 s) conditions. Furthermore, the main effect for the testing time (F_(2,32)_ = 65.93, *p* < 0.001, η_p_^2^ = 0.805) showed a significant underestimation of time in the follow-up (MD = −1.308 s) and post-test (MD = −1.244 s) compared to the pre-test (Table 2).

#### 5.1.3. Twenty Seconds

Furthermore, the 20 s time estimates revealed a significant main effect for fatigue conditions (F_(1.35,21.64)_ =17.12, *p* = 0.001, η_p_^2^ = 0.517), testing time (F_(1.44,23.03)_ = 4.27, *p* = 0.037, η_p_^2^ = 0.211), and as well as for the interaction between testing time and conditions (F_(4,64)_ = 17.12, *p* = 0.001, η_p_^2^ = 0.519). There was a significant, large-magnitude underestimation of time in the interaction between condition and testing time for physical fatigue compared to mental fatigue (MD = −3.418 s, *p* < 0.001, d = 2.11) and control (MD = −2.708 s, *p* < 0.001, d = 2.39) conditions at the post-test and mental fatigue (MD = −3.726 s, *p* < 0.001, d = 1.93) and control (MD = −2.976 s, *p* < 0.001, d = 2.13) conditions in the follow-up, but there were no significant interactions between condition and testing time at the pre-test among conditions. The main effect for conditions revealed a significant underestimation of time with the physical fatigue condition compared to mental fatigue (MD = −2.436 s, *p* < 0.001) and control (MD = −2.093 s, *p* < 0.001) conditions, but there were no significant differences between mental fatigue and control conditions. The main effect for the testing time demonstrated a significant underestimation of time in the follow-up (MD = −2.314 s) and post-test (MD = −2.376 s) compared to the pre-test, but there were no significant differences between the post-test and follow-up (Table 2).

#### 5.1.4. Thirty Seconds

The 30 s time estimates showed a significant main effect for fatigue conditions (F_(1.28,20.58)_ = 15.65, *p* = 0.001, η_p_^2^ = 0.495) and testing time (F_(1.27,20.46)_ = 5.01, *p* = 0.029, η_p2_ = 0.239), as well as for the interaction between testing time and conditions (F_(4,64)_ = 15.90, *p* = 0.001, η_p_^2^ = 0.499). The interaction between condition and testing time revealed that there were no significant differences between conditions at the pre-test, but there was a significant, large-magnitude underestimation of time with physical fatigue compared to mental fatigue (MD = −4.763 s, d = 1.89) and control (MD = −3.829 s, d = 2.45) conditions at the post-test and mental fatigue (MD = −5.218 s, d = 1.82) and control (MD = −3.700 s, d = 2.01) conditions in the follow-up. Additionally, there were no significant differences between mental fatigue and control conditions at the pre-test, post-test, or follow-up. The main effect for conditions revealed an underestimation with physical fatigue compared to mental fatigue (MD = −3.458 s, *p* < 0.001) and control (MD = −2.786 s, *p* < 0.001) conditions, but there were no significant differences between mental fatigue and control conditions. A significant main effect for the testing time showed a significant underestimation of time in the follow-up (MD = −3.208 s) and post-test (MD = −3.399 s) compared to the pre-test, but there were no significant differences between the post-test and follow-up (Table 2).

### 5.2. Relative (%) Time Changes between Each Time Estimate

**Figure 2:**
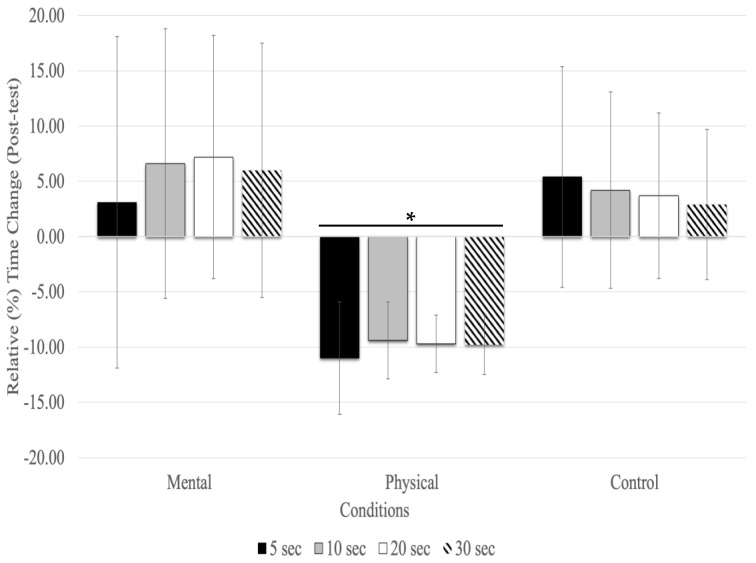
Relative (%) time change for the time estimates at the post-test (mean % ± SD). Only the physical fatigue condition resulted in a significant (asterisk and horizontal line represent *p* < 0.01) underestimation of time.

With the post-test, relative time changes showed a significant main effect of conditions (F_(1.27,20.43)_ = 16.83, *p* = 0.001, η_p2_ = 0.513) and testing time (F_(1.36,21.89)_ = 4.12, *p* = 0.044, η_p_^2^ = 0.205), but there was no significant interaction between testing time and conditions (F_(2.60,41.63)_ = 0.628, *p* = 0.579, η_p_^2^ = 0.038). The main effect for fatigue conditions showed that physical fatigue resulted in a significant relative underestimation of time compared to mental fatigue (MD = −0.158 s, *p* < 0.001) and control (MD = −0.129 s, *p* < 0.001) conditions. There was no significant relative time change between time estimates (5, 10, 20, and 30s) at the post-test with all conditions combined (Figure 2).

**Figure 3:**
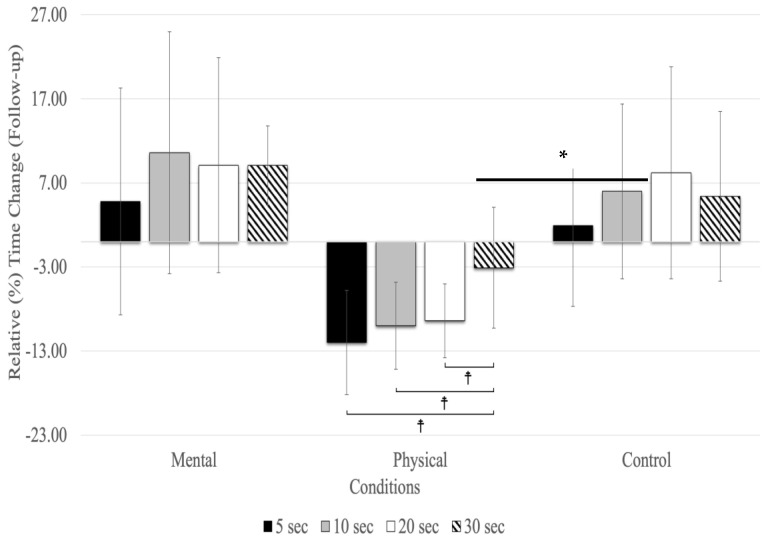
Relative (%) time change for the time estimates at the follow-up (mean % ± SD). Only the physical fatigue condition resulted in a significant (asterisk and horizontal line represent a main effect for the condition: *p* < 0.001) underestimation of time. ☨ indicates *p* < 0.001 compared to the other time estimates.

At the follow-up, relative time changes showed a significant main effect for testing time (F_(1.72,27.53)_ = 3.70, *p* = 0.018, η_p_^2^ = 0.188) and conditions (F_(2,32)_ = 18.09, *p* = 0.001, η_p2_ = 0.531), as well as for the interaction between testing time and conditions (F_(2.32,27.22)_ = 25.01, *p* = 0.001, η_p_^2^ = 0.610) (Figure 3). The main effect for testing time (all conditions combined) revealed relative time changes between 5, 10, 20, and 30 s in the follow-up, with 10 (MD = 0.040 s, *p* = 0.005) and 20 s (MD = 0.044 s, *p* = 0.039) time estimates showing significantly higher overestimates than 5 s (F_(1.72,27.53)_ = 3.70, *p* < 0.043, η_p_^2^ = 0.188). There were no significant differences between 30 s and the other time estimates (5, 10, and 20 s) in the follow-up testing period (Figure 3). The main effect for conditions showed that the relative time changes due to physical fatigue were significantly underestimated compared to mental fatigue (MD = −0.110 s, *p* = 0.002) and control (MD = −0.125 s, *p* = 0.001) conditions. The interaction between testing time and conditions revealed relative time changes, showing that due to mental fatigue, 30 s was underestimated compared to the 5 (MD = −0.140 s, *p* = 0.005), 20 (MD = −0.183 s, *p* < 0.001), and 10 s (MD = −0.198 s, *p* < 0.001). The results of relative time changes for physical fatigue revealed that 30 s was overestimated compared to 20 (MD = 0.126 s, *p* < 0.001), 10 (MD = 0.132 s, *p* < 0.001), and 5 (MD = 0.152 s, *p* < 0.001) s. Additionally, there was no significant relative time change for the control condition.

## 6. Heart Rate

**Figure 4:**
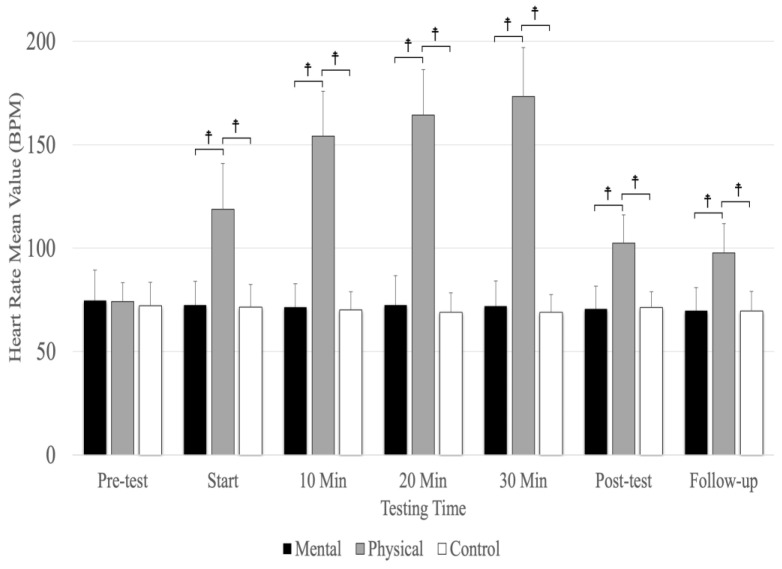
The means and standard deviations of heart rate for the three conditions (physical fatigue, mental fatigue, and control) at the seven testing times. ☨ indicates *p* < 0.001 compared to the control and mental fatigue conditions.

Analyses revealed a significant main effect for the testing time (F_(3.69,59.09)_ =193.12, *p* = 0.001, η_p_^2^ = 0.923) and conditions (F_(1.42,22.77)_ =286.40, *p* = 0.001, η_p_^2^ = 0.947) as well as a significant interaction between testing time and conditions (F_(4.23,67.73)_ =158.18, *p* = 0.001, η_p_^2^ = 0.908). There were no significant differences between conditions at the pre-test, but physical fatigue (*p* < 0.001) resulted in a significantly higher heart rate compared to the mental fatigue and control conditions at the start; 10, 20, and 30 minutes; post-test; and follow-up (Figure 4). The main effect for conditions showed a significant, large-magnitude increase in heart rate due to physical fatigue compared to the mental fatigue (MD = 54.513, *p* < 0.001) and control (MD = 56.126, *p* < 0.001) conditions. There was no significant difference between mental fatigue and control conditions. The main effects for testing time showed a significant difference (*p* < 0.001) between the pre-test; start; 10, 20, and 30 min; and follow-up, except for 20 and 30 min (*p* = 0.184) and between the post-test and follow-up (*p* = 0.207) (Figure 4).

## 7. Tympanic Temperature

**Figure 5:**
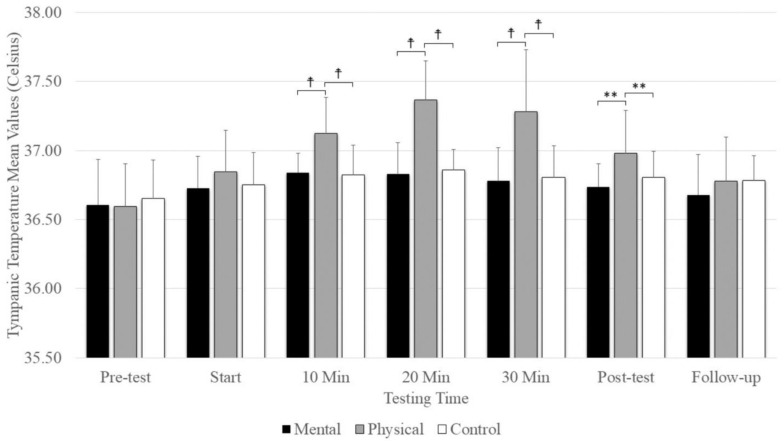
The means and standard deviations of tympanic temperature under the three conditions (physical fatigue, mental fatigue, and control) at the seven testing times. ☨ indicates *p* < 0.001 compared to the control and mental fatigue conditions. ** indicates *p* < 0.01 compared to the control and mental fatigue conditions.

A significant main effect was evident for testing time (F_(2.74,43.84)_ = 27.18, *p* = 0.001, η_p_^2^ = 0.629) and conditions (F_(2,32)_ =13.35, *p* = 0.001, η_p_^2^ = 0.455), as well as the interaction between testing time and conditions (F_(4.70,75.32)_ = 8.925, *p* = 0.001, η_p_^2^ = 0.358). There were no significant differences between conditions at the pre-test and start, but there was significantly elevated tympanic temperature with physical fatigue (*p* = < 0.001) compared to mental fatigue and control conditions at 10, 20, and 30 min and at the post-test (Figure 5). There was no significant difference among conditions at follow-up. The main effect for conditions showed that physical fatigue resulted in a significant, large-magnitude increase in tympanic temperature compared to mental fatigue (*p* = 0.001) and control (*p* = 0.008) conditions. The main effects for testing time showed a significantly higher tympanic temperature difference at the pre-test (*p* < 0.001) compared to the start; 10 min, 20 min, and 30 min; post-test; and follow-up (Figure 5).

## 8. Rating of Perceived Exertion (RPE)

**Figure 6:**
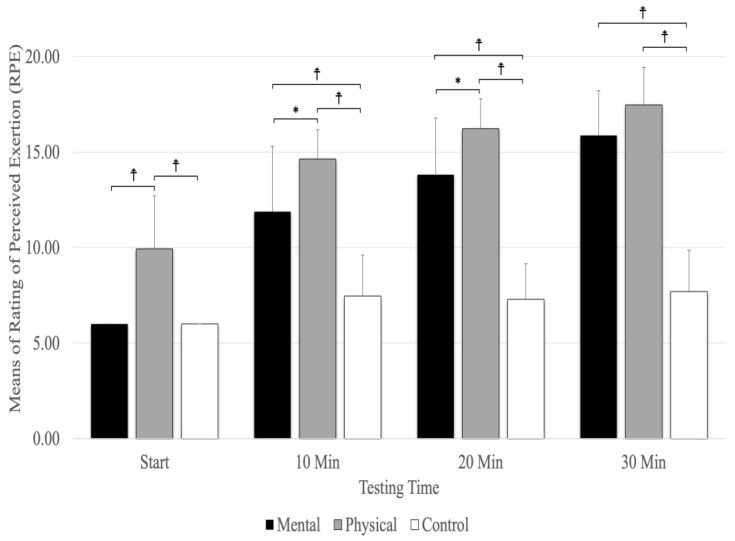
The means and standard deviations of Rating of Perceived Exertion (RPE). ☨ indicates *p* < 0.001 compared to the control and mental fatigue conditions. * Indicates *p* < 0.05 compared to mental fatigue condition.

A significant main effect was evident for testing times (F_(1.78,28.51)_ =170.51, *p* = 0.001, η_p_^2^ = 0.914) and conditions (F_(2,32)_ =106.80, *p* = 0.001, η_p_^2^ = 0.870), as well as the interaction between testing times and conditions (F_(3.04,48.63)_ = 24.72, *p* = 0.001, η_p_^2^ = 0.607). The interaction between condition and testing time showed that physical fatigue (*p* < 0.001) resulted in a higher RPE than mental fatigue and control conditions at the start and at 10 and 20 min (Figure 6). The results revealed that physical fatigue and mental fatigue (*p* < 0.001) resulted in a higher RPE than the control conditions, but there were no significant differences between physical fatigue and mental fatigue (*p* = 0.157) at 30 min. The main effect for the fatigue conditions showed significantly higher RPE scores for physical fatigue versus mental fatigue and control conditions (*p* < 0.001). The main effects for testing time showed a significant difference between RPE (*p* < 0.001) at the start and 10, 20, and 30 min (Figure 6).

## 9. The NASA Task Load Index

### 9.1. Mental Demand

One-way repeated measures ANOVA (F_(2,32)_ = 41.87, *p* < 0.001, η_p_^2^ = 0.724) revealed that the mental fatigue condition had a large-magnitude mental demand compared to physical fatigue and control conditions. Additionally, there were no significant differences between physical fatigue and control conditions (Table 3).

### 9.2. Physical Demand

Physical fatigue had a large-magnitude, significant (F_(1.23,19.69)_ = 167.241, *p* < 0.001, η_p_^2^ = 0.913), and higher physical demand compared to mental fatigue and control conditions, but there were no significant differences between mental fatigue and control conditions (Table 3).

## 10. Discussion

To the best of our knowledge, this is the first study to compare the impacts of mental and physical fatigue on the perception of time. The major findings of this research revealed that participants subjected to physical fatigue exhibited a significant underestimation of time intervals during the post-test and follow-up when compared to those subjected to the mental fatigue and control conditions. In addition, physical fatigue resulted in a significantly higher, relative (%) underestimation of time change in comparison to mental fatigue and control conditions. Moreover, physical fatigue induced significantly higher tympanic temperatures and heart rates during the intervention and post-test compared to the mental fatigue and control conditions. The NASA Task Load Index demonstrated the efficacy of both the physical and mental fatigue protocols in inducing states of physical and mental fatigue.

The underestimations of the 5, 10, 20, and 30 s time estimates with the physical fatigue condition are in line with the hypothesis predicting significant time underestimations (estimated time was shorter than chronological time) compared to the mental fatigue and control conditions. Moreover, the results for physical fatigue (an underestimation of time) are congruent with Graham et al. [15], as their results showed an underestimation of time across three exercise conditions (30 s of knee extensor exercises at 100%, 60%, and 10% of maximum voluntary isometric contraction) with all time estimates (5, 10, 20, and 30 s) compared to the control condition. In addition, the present study’s findings are generally consistent with Gardner et al. [29], who revealed that maximal contractions induced significantly greater time underestimations at 5, 20, and 30 s than the control conditions. Moreover, their study showed that submaximal contractions (60% of maximal voluntary isometric contractions) also contributed to time underestimation at 30 s. Furthermore, Edwards and McCormick [40] utilized cycling, wherein participants were asked to estimate the completion of 25%, 50%, 75%, and 100% of the trial duration under various RPE conditions. Notably, they observed that at the 75% and 100% intervals, time estimates for the RPE 20 condition, representing maximal exertion, exhibited the shortest durations when compared to those of RPE 11 (light intensity) and RPE 15 (moderate intensity). Additionally, the participants also completed a rowing task, wherein they found similar intensity-dependent results [40]. Similarly, the RPE findings indicated that participants, upon the end of the physical fatigue intervention, reported an average RPE score of 17. This finding aligns with a study by Edwards and McCormick [40], who suggested that the perceived level of exertion experienced during the physical fatigue condition might be an indicator of the underestimation of time in that physical fatigue condition.

In some studies, it has been suggested that an increase in body temperature affects temporal perception [41,42]. Although there is conflicting evidence on whether tympanic temperature is a good index of core and brain temperature [43,44], two studies have shown that core temperature increased with running in a warm, humid environment, corresponding to an underestimation of time [26,27]. Similarly, in the present study, the tympanic temperature was significantly higher in the physical fatigue condition compared to mental fatigue and control conditions during the intervention and the post-test. This finding diverges from Graham et al.’s [15] study, which reported an absence of a significant increase in tympanic temperature. Similarly, Gardner et al. [29] showed that tympanic temperature remained unaffected by the contraction intensities. One possible reason for the observed disparity in outcomes between the present study and the prior investigation lies in the dissimilarities in methodological approaches. Notably, they employed isometric contraction as the primary exercise modality, while we opted for 30 min cycling at 65% PPO, which induced a greater rise in tympanic temperature compared to Graham et al.’s [15] and Gardner et al.’s [29] studies. Moreover, it is pertinent to acknowledge that the duration of their experimental protocol was comparatively shorter than ours, which may have further contributed to differences in physiological reactions and the subsequent findings of the two studies.

Another notable finding in this study pertains to the heart rate, which exhibited a significant elevation during the physical fatigue condition at the stages of intervention, post-test, and follow-up in comparison to both the mental fatigue and control conditions. This finding aligns with the findings of Gardner et al. [29], who reported lower heart rate values for the control condition (75.3 ± 11.6) in contrast to the maximal (92.5 ± 13.9), 60% submaximal (92.2 ± 14.4), and distraction (90.5 ± 14.7) conditions. Similarly, the results obtained by Graham et al. [15] are consistent with our study, as they demonstrated that the control condition exhibited lower heart rate values (beats per minute) (74.6 ± 10.6) compared to the maximal (91.6 ± 12.4), 60% MVIC (92.5 ± 13.8), and 10% MVIC (90.7 ± 13.5) conditions.

Contrary to our initial hypothesis, the results of our analysis of the mental conditions do not align with our hypothesis, as participants did not exhibit a tendency to underestimate the perception of time in this condition. The ideal task duration to induce mental fatigue in young adults is currently unclear in the literature; thus, it is not known how long it takes for a change in task performance to become significant. Previous studies have employed tasks that continue for several hours. However, new research indicates that 30 to 90 min is sufficient to cause mental fatigue [45]. Individual differences may also be a significant factor in the onset of mental fatigue and the length of time required to induce it. Evidence from some studies suggests that shorter task durations may be sufficient to cause mental fatigue, as significant declines in cognitive function were seen after only 30 min [36], 45 min [45], 60 min [46], and 90 min [47]. In their study, Vrijkotte et al. [48] used a 90 min Stroop task to induce mental fatigue. The primary conclusion of their research is that in trained, young, healthy athletes, a large magnitude of mental fatigue, as determined by the NASA Task Load Index, had no impact on physical or cognitive performance (accuracy and reaction times) during the second exercise bout of the two-bout exercise protocol [48]. They found that when no mentally fatiguing task was being conducted, the initial maximal exercise test also increased mental fatigue. The individuals were unable to distinguish between physical and mental fatigue [48]. One study showed that performing the Stroop task for 30 min successfully induced mental fatigue [36]. Although the NASA-TLX showed that the Stroop task induced mental fatigue for the participants, the 1100 trials (approximately 30 min) might not have been enough to affect time perception or had a shorter duration of impact after the mental fatigue protocol. Another possible mechanism is that mental fatigue and physical fatigue might have different physiological and psychological mechanisms that affect time perceptions differently. Our findings for the physical fatigue condition are not consistent with Tonelli et al.’s [28] study, which investigated the effects of moderate physical activity (cycling) on a temporal estimation task in a group of adult volunteers under three different conditions: (1) baseline, (2) during the physical activity phase, and (3) roughly 15 to 20 min later, when participants were seated and returned to a resting heart rate (POST). They discovered that exercise directly alters how people perceive time, causing them to overestimate durations in the millisecond range. Notably, the impact lasted during the POST session, ruling out either heart rate or cycle rhythmicity as the primary contributors [28].

It was anticipated that when participants estimated the four successive times (5, 10, 20, and 30 s), gradually, time variability would increase. Naturally, you would anticipate more time variability as time goes on because minor time estimate errors made early in the trial can become more amplified as time goes on [15]. However, it was intriguing that the relative results showed that 5, 10, and 20 s intervals demonstrated relatively higher underestimations of time for the physical fatigue condition compared to 30 s.

The findings that physical fatigue can lengthen an individual’s subjective experience of time can be elucidated from the perspective of the Pacemaker–Accumulator Model (PAM), as posited by [6,7,10]. Specifically, under the physical fatigue condition, participants were cycling at 65% of PPO. This physical exertion induced muscle fatigue and discomfort attributed to factors such as tension, partial blood occlusion, and metabolite accumulation, among others. This adverse sensation functions as a type of physiological arousal [49]. Arousal has been found to elevate the speed of the pacemaker, resulting in an increased number of pulses accumulated in the accumulator [50,51]. This heightened arousal contributes to a perceived distortion of time, leading to a specific lengthening of perceived time intervals [50]. Importantly, this time distortion effect exhibits a multiplicative characteristic, wherein the extent of distortion intensifies with longer stimulus durations [52]. Consequently, it is plausible to hypothesize that arousal induced by exercise could engender a time distortion effect [4,53]. In the present study, the heightened state of arousal post-exercise contributed to the sustained increase in heart rate and body temperature during the post-test phase. This suggests that the distortion in the perception of time persists even after the cycling activity has concluded. The study findings indicate that mental fatigue led to a slight overestimation of time when compared to chronological time; however, the observed difference did not reach statistical significance. Possible reasons for this outcome can be attributed to the limited number of trials employed in the Stroop task, which might not have been sufficient to induce a notable distortion in time perception. Additionally, it is plausible that mental fatigue and physical fatigue operate through distinct mechanisms, which could contribute to differential effects on the perception of time. Further investigation and a more comprehensive experimental design are warranted to delve deeper into these intricacies and better comprehend the underlying factors influencing temporal perception in the context of mental and physical fatigue.

## 11. Limitations

This research investigation, akin to many other studies, was not devoid of limitations. One of the hypotheses of the study aimed to compare time estimates between male and female cohorts. However, due to challenges in recruiting an adequate number of female participants (3/17), this objective remained unfulfilled. Consequently, the sample primarily consisted of male students engaged in recreational physical activities. An additional limitation of this study pertains to the substantial standard deviations in relation to the mean values, reflecting considerable heterogeneity among the various individual outcomes. Future studies should investigate how mental and physical fatigue might affect the perception of time in males and females differently. Additionally, how might time perception be different for endurance exercise above the lactate threshold (e.g., >80–85% max aerobic power)?

## 12. Practical Applications

The present study suggests that individuals engaged in physically demanding activities, such as sports, driving, and work settings, among others, may experience alterations in time perception due to the influence of physical fatigue. Accordingly, it is recommended that these individuals engage in deliberate exercises aimed at enhancing their time perception abilities during periods of physical fatigue. Such practices are hypothesized to facilitate the development of an enhanced sense of timing under physically demanding conditions.

Furthermore, there is another practical aspect to consider: how can we offer feedback or modify exercise routines for individuals who perceive themselves as having limited time available for improvement in order to enhance adherence? This question holds significant relevance, particularly for the general populace that may not find exercise enjoyable. Moreover, the prospective time estimation ratio could also have a notable influence on endurance athletes or time-restricted athletes (e.g., tennis, basketball, and North American football) who require precise pacing or timing. This factor carries substantial implications, as an athlete who underestimates the time may perform too slowly, jeopardizing their chances of winning a race, whereas an overestimation of time might lead them to push too hard and experience fatigue.

## 13. Conclusions

This study’s findings highlight the impact of physical and mental fatigue on participants’ perceptions of time. Specifically, under the physical fatigue condition, participants underestimated time during the post-test and follow-up, as compared to the mental fatigue and control conditions, across various time intervals (5, 10, 20, and 30 s). Moreover, the investigation revealed no significant differences between the mental fatigue and control conditions concerning time estimates. In addition, the results showed that the physical fatigue condition resulted in significantly higher RPE, heart rates, and body temperatures, hence the greater physical demand under the physical fatigue condition as compared to the mental fatigue and control conditions. Additionally, mental demand was significantly higher in the mental fatigue condition than in the physical fatigue and control conditions.

## Figures and Tables

**Table 1:** Participant anthropometrics.

Participants	Age (Years)	Mass (kg)	Height (cm)
Male (*n* = 14)	28.57 ± 4.92	80.46 ± 11.22	175.71 ± 2.65
Female (*n* = 3)	24 ± 2.64	69.06 ± 10.96	157.33 ± 2.30

**Table 2:** Means and standard deviations of the time estimates of 5, 10, 20, and 30 s from the chronological time at the pre-test, post-test, and follow-up. Asterisks (*) illustrate significant differences from chronological time.

Time Estimates	Mental Fatigue (M ± SD)	Physical Fatigue (M ± SD)	Control (M ± SD)
***Deviation from chronological time for 5 s estimates***			
Pre-test	−0.059 ± 0.416	−0.011 ± 0.288	0.121 ± 0.478
Post-test	0.155 ± 0.751	−0.550 ± 0.255 *	0.026 ± 0.500
Follow-up	0.240 ± 0.677	−0.601 ± 0.314 *	0.096 ± 0.483
***Deviation from chronological time for 10 s estimates***			
Pre-test	0.360 ± 0.734	0.301 ± 0.549	0.555 ± 0.927
Post-test	0.669 ± 1.228	−0.942 ± 0.350 *	0.423 ± 0.898
Follow-up	1.061 ± 1.446	−1.006 ± 0.524 *	0.603 ± 1.040
***Deviation from chronological time for 20 s estimates***			
Pre-test	0.581 ± 1.154	0.417 ± 1.136	1.014 ± 1.542
Post-test	1.458 ± 2.219	−1.958 ± 0.538 *	0.748 ± 1.502
Follow-up	1.830 ± 2.570	−1.896 ± 0.898 *	0.748 ± 1.502
***Deviation from chronological time for 30 s estimates***			
Pre-test	0.846 ± 1.938	0.452 ± 1.733	1.281 ± 2.322
Post-test	1.815 ± 3.459	−2.947 ± 0.839 *	0.881 ± 2.042
Follow-up	2.461 ± 3.790	−2.756 ± 1.413 *	0.943 ± 2.175

**Table 3:** The means and standard deviations (SDs) for the mental and physical demand. Asterisks indicate that the highlighted condition was significantly (*p* < 0.01) greater than the other two conditions.

Conditions	Mental Demand Mean ± SD	Physical Demand Mean ± SD
Mental fatigue	80 ± 18.28 *	14.41 ± 14.45
Physical fatigue	34.11 ± 20.63	81.17 ± 14.09 *
Control	23.23 ± 25.18	11.67 ± 17.31

## Data Availability

Data is available upon request.
